# Stimulating *Duddingtonia flagrans* chlamydospore production through dehydration

**DOI:** 10.1007/s00436-019-06499-0

**Published:** 2019-11-18

**Authors:** Justin Blair, Amy Biddle

**Affiliations:** College of Agriculture and Natural Resources, University of Delaware, 034 Townsend Hall, Newark, DE 19716 USA

**Keywords:** *Duddingtonia flagrans*, Helminth, Biological control, Chlamydospore production

## Abstract

*Duddingtonia flagrans* is a nematode-trapping fungus that has shown promising results as a tool to combat parasitic nematode infections in livestock. The fungus interrupts the parasitic lifecycle by trapping and killing larval stages on pasture to prevent re-infection of animals. One barrier to the fungus’ commercial use is scaling up production of the fungus, and specifically of chlamydospores, which survive the digestive tract to grow in fecal pats on pasture, thus have potential as a feed through anthelmintic. The purpose of this study was to evaluate the effect of dehydration on sporulation of the fungus. Disks of *Duddingtonia flagrans* type strain (ATCC® 13423™) were grown on 17% cornmeal agar for 26 days at 30 °C, then split into three groups; dried quickly at 38 °C and 37% humidity over 48 h (“incubated”), dried more slowly at 24 °C and 55% humidity over 10 days (“air-dried”), or kept at 30 °C and sealed with parafilm to prevent loss of moisture as a control (“wet”). Half of each dried culture was resuspended in water*,* then heated to liquify and homogenized through vortexing. Spores were then counted in a Neubauer hematocytometer. Both the “air-dried” and “incubated” drying techniques yielded significantly more spores than the “wet” control (Welch’s two sample *t* test *p* values of .0359 and .0411, respectively). The difference in average chlamydospores per milliliter was insignificant between the two drying techniques, although a visual representation of the data shows less spore count variability in the “air-dried” technique.

## Introduction

As anthelminthic drug resistance continues to increase in parasitic nematode populations around the globe, alternative treatment methods and control regimens continue to gain importance. *Duddingtonia flagrans* is a predatory fungus that forms trapping structures in the presence of nematodes, capturing and digesting them. The ability of *D. flagrans* to grow in fecal pats, persist on pasture, then capture, and kill larval stages of nematodes, makes it an attractive biological control agent. It targets parasites in the environmental life cycle stage, thereby preventing infection. In addition, *D. flagrans* forms chlamydospores that are capable of surviving the digestive tract of host animals (Larsen et al. 1992; Larsen 1999; Grønvold et al. 1993; Faedo et al. 1997; Ojeda-Robertos et al. 2009), delivering the fungus directly to the fecal pats where the larvae will hatch. This allows it to be used as a feed additive, which is a more convenient and efficient treatment method than broadcast spreading over pasture. The fungus has a minimal environmental impact and a broad range of potential targets in both ruminant and non-ruminant host species (Braga and Araújo 2014).

A few limitations of the use of *D. flagrans* include the necessity of cell-cell signaling in trap formation and constriction that requires hyphal fusion and a significant mycelial growth to achieve a switch from saprotrophic to zootrophic nutrition intake (Youssar et al. 2019). This denotes a requirement of a certain threshold chlamydospore administration to cause sufficient growth for this transformation. Another potential limitation is the induction of trap formation. The fungus has been shown to increase sensitivity to nematode excreta in prolonged starvation conditions (Anan’ko and Teplyakova 2011), so a processing step that includes starvation of the fungus may be beneficial to its use as an anti-parasitic treatment.

Even with the fungus’s ability to be used as a direct feed additive, large-scale production of the fungus has been one of the major limiting factors restricting its use commercially (Santurio et al. 2009). Previous studies have examined the effects of growing the fungus in different solid and liquid culture mediums, such as measuring the growth rates in shake flask media types (Gardner et al. 2000), use of a two-step liquid/solid technique using sterile grain (Santurio et al. 2009), and measuring the spore production rates using different growth inducing media additives and temperatures (Sagüés et al. 2012), all in an effort to discover how to optimize fungus and fungal chlamydospore production. Chlamydospores are an adaptation that evolved to survive harsh conditions, where nutrients are shunted to a thick-walled specialized hyphal segment. They are also sometimes referred to as resting spores and are thought to be the portion of the fungus that survives digestion (Ojeda-Robertos et al. 2009).

Although studies have been performed on optimizing growth of chlamydospores during fungal hyphal growth, examination of the effects of altering environmental conditions on existing cultures is understudied. The objective of this experiment was to quantify the effect of fast drying the fungus (high temperature and low humidity) compared with slow drying (low temperature and high humidity) on the production of chlamydospores compared with control cultures that were continuously left in moist conditions. This could provide a method of pushing previously defined limits in chlamydospore production and aid in *D. flagrans* use as a control for parasitic infections in grazing animals.

## Materials and methods

### Fungal strain

For this study, the strain used was *D. flagrans* (ATCC® 13423™), strain designation CBS 565.50 [IMI 101314], originally isolated from vegetable compost in England (Duddington 1949). Initial culture material was ordered from the American Type Culture Collection and reconstituted then maintained by continuous culture transfer. *D. flagrans* identity was confirmed using MO BIO PowerFecal DNA extraction kit, followed by PCR amplification of the ITS2 genomic region using the primers ITS86F—GTGAATCATCGAATCTTTGAA and ITS4—TCCTCCGCTTATTGATATGC (Vancov and Keen 2009). PCR product was sequenced using Sanger sequencing at the University of Delaware DNA Sequencing & Genotyping center at the Delaware Biotechnology Institute; sequence was trimmed and blasted against the NCBI database using Geneious® software (Kearse et al., 2012).

### Fungal cultures

*D. flagrans* cultures were grown on BBL™ Corn Meal Agar (BD Biosciences, Sparks, MD) 17 g/L with no media additives or supplements. Two incubation chambers were used to maintain 30 °C and 38 °C temperatures, and 24 °C was ambient room temperature. A mobile AcuRite® (Chaney Instrument Co., Lakek Geneva, WI) humidity detector was used to monitor humidity for the two unsealed culture groups.

A total of 12 cultures were grown in sealed plates at 30 °C for 26 days, inoculated by media transfer from the same established growth plate. These were randomly sorted in to 3 groups of 4 each. One group, the quick drying “incubated” treatment, was uncovered and dehydrated at 38 °C and 37% humidity. The second group, the slower drying “air-dried” treatment, was uncovered and dehydrated at 24 °C and 55% humidity. The third group, the “wet” control group, was kept sealed and left at 30 °C until collection and spore counting as a control.

The control cultures weighed on average 2.4 g. Half of each control culture was collected (average 1.16 g) and added to a 2-mL Eppendorf tube and heated to 95 °C for 90 min vortexing for 20 s every 30 min to liquify and homogenize the solution. The heated solution was sampled twice for each culture and quickly loaded into a hemocytometer and observed in phase contrast for spore counting.

The fully dried cultures in both of the “incubated” and “air-dried” treatment groups had an average weight of 0.1 g. Half of each dry culture was collected (~ 0.05 g) and added to a 2-mL Eppendorf tube along with 1200 μL of Milli-Q purified water to equalize the weight/volume of the control samples, heated for 90 min at 95 °C with vortexing to homogenize (as described above), and then quickly loaded into a hemocytometer for spore counting, two samplings from each culture. Counts were verified following 12 h of heating at 95 °C (to fully denature the agar) with vortexing as described above. The spore counts were conducted using all 9 counting grids in the hemocytometer and converted to spores per milliliter by averaging and then dividing by the known volume of each grid (0.0001 mL) and rounded to the nearest whole spore.

Thirty days after the conclusion of the study, a subsample of each culture was added to a sterile cornmeal agar plate and observed to confirm viability of the spores. Two milliliters of the liquified media used for spore counting was also plated and observed to determine if any spores had remained viable after continued exposure to extreme heat (95 °C).

### Statistical analysis

One-tailed Welch two sample *t* tests were performed in R (R Core Team 2016) using R Studio (R Studio Team 2016) to compare the population means of numbers of spores per milliliter for each culture group to controls, taking into account the unequal variances in the sample groups. Data visualizations were also produced in (R Core Team 2016) using R Studio (R Studio Team 2016).

## Results

**Fig. 1:**
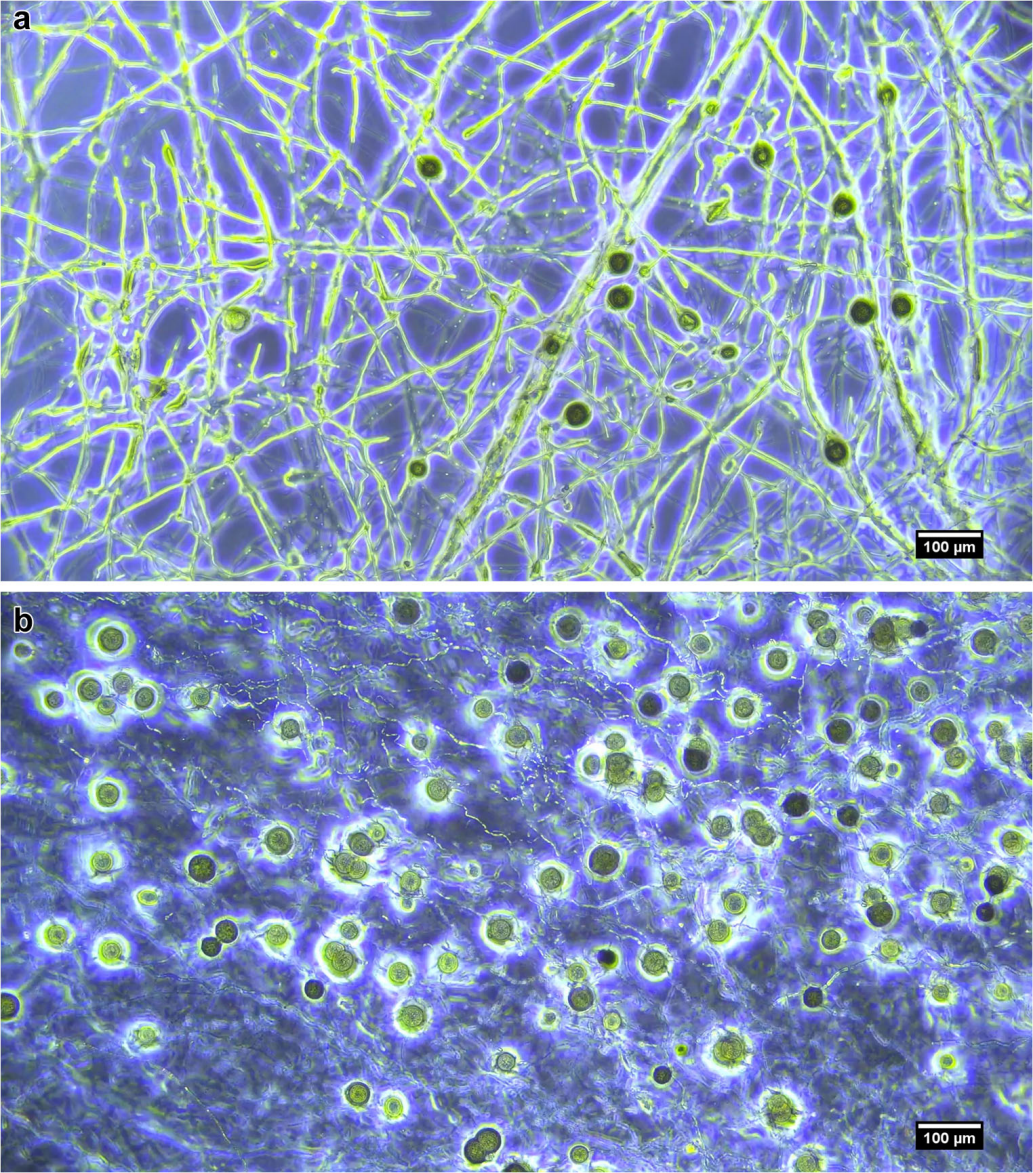
**a** Sample of *Duddingtonia flagrans* kept in wet conditions on 17% cornmeal agar. **b** Sample of *Duddingtonia flagrans* grown on 17% cornmeal agar and subsequently dehydrated. The difference in media volume is approx. 92% reduction between **a** and **b**

The fungal DNA extracted and sequence to confirm the identity of the fungus had a post-quality trimmed read length of 318 bp and 100% match to Duddingtonia flagrans partial sequence, E value of 3.43 × 10^−164^. The “incubated” treatment cultures that were dehydrated at 38 °C and 37% humidity appeared completely dehydrated after 48 h, and the “air-dried” cultures dehydrated at 24 °C and 55% humidity appeared completely dehydrated after 10 days. Figure 1 shows a visual example of the difference between a wet control culture and a dry treatment culture, although these images are not quantitative and the difference in volume should be taken into consideration between the two images with a 92% reduction in volume from the wet sample to the dry sample. It is also evident that the normal hyphal growth has been extremely dehydrated to the point of destruction in the dry sample, but the relative size of the chlamydospores remains similar as they are resistant to dehydration; an important factor in their use as a biological control agent for parasitic infections post-dehydration.

Wet sample number 2 in the control group had a crack in the parafilm seal and dried to a weight of .25 g, compared with the other wet samples at an approximate weight of 2.5 g. This culture was still sampled but the data was excluded from analysis because of its unique conditions. The two sampling methods used were sampling immediately after heating and vortexing for the first two samplings from each group, and then sampling after 12 h of 95 °C and vortexing for the third sampling in each group. A Welch two sample *t* test between the third sampling and each of the first two samplings for the respective treatment groups did not show a significant difference so all three samplings were used in data analysis. Weights of the culture material used before the addition of water to normalize the volumes can be seen in Table 1.

**Table 1:** Weight of each culture collected for counting after drying treatment, wet corresponds to control cultures, air to 24 °C drying group, and Inc. to 38 °C drying group

	Sample 1 (g)	Sample 2 (g)	Sample 3 (g)	Sample 4 (g)
Wet	1.23	0.12	0.99	1.27
Air	0.06	0.05	0.05	0.06
Inc.	0.06	0.06	0.08	0.05

**Fig. 2:**
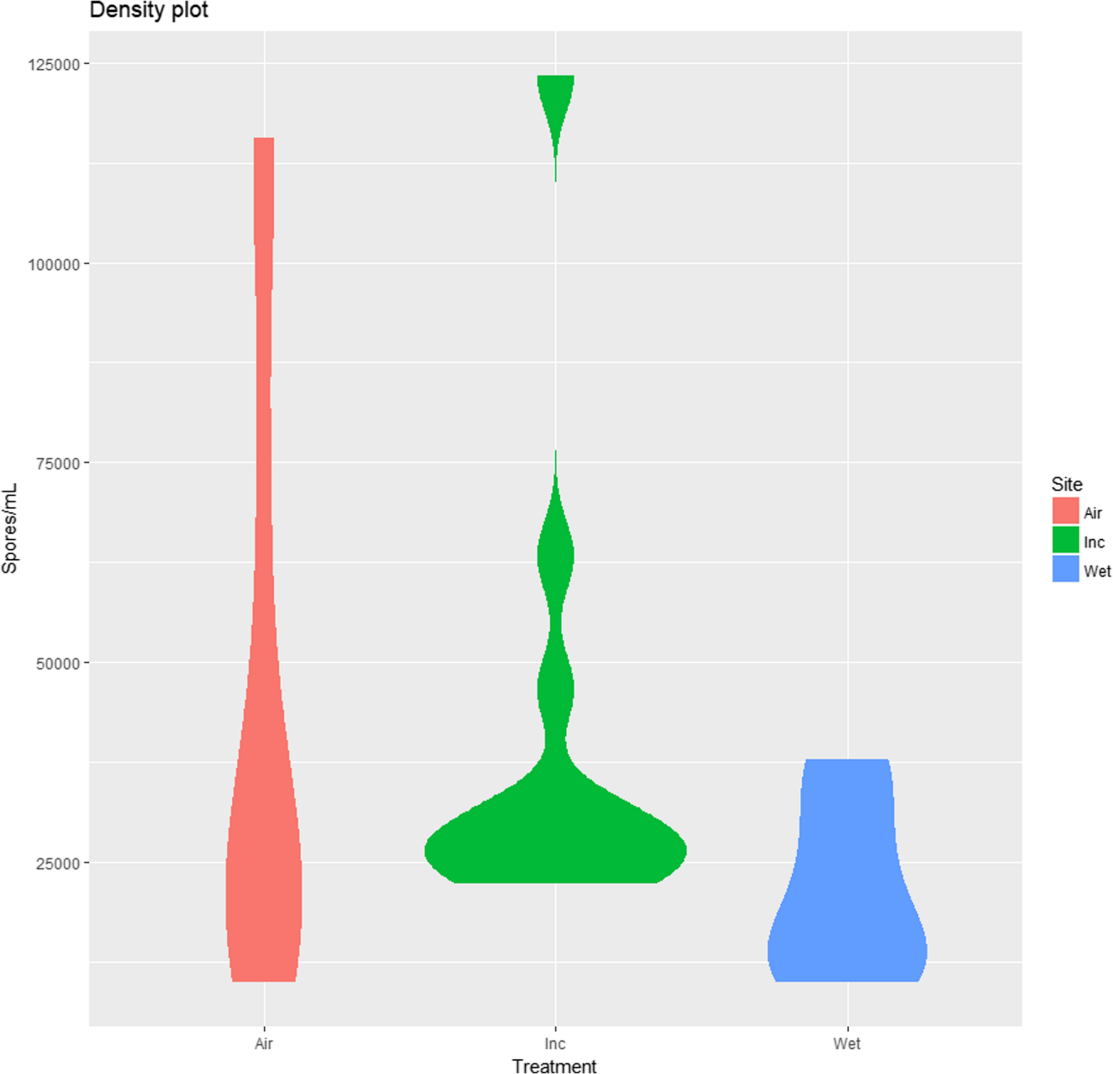
Violin density plot of number of spores per milliliter, with air corresponding to 24 °C drying group, Inc. to 38 °C drying group, and wet to control cultures that were kept moist

Both of the dried treatment culture groups had significantly more spores (*p* < .05) than the control cultures with averages of 42,780 spores/mL for the “incubated” treatment group and 39,260 spores/mL for the “air-dried” treatment group. The “wet” control group had an average of 21,480 spores/mL. The spore counts can be seen in Table 2. A Welch two sample *t* test shows the difference in means between the two drying treatments is insignificant (*p* = .80). The slower “air-dried” drying method had a lower mean number of spores, and lower maximum and minimum spores measured, but produced more consistent measurements than the quicker “incubated” drying method as seen in the violin density plot Fig. 2.

**Table 2:** Number of spores per mL after conversion from Neubauer hemocytometer counting chamber, with wet corresponding to control cultures, air to 24 °C drying group, and Inc. to 38 °C drying group

	Count 1	Count 2	Count 3
Wet 1	37,778	37,778	24,444
Wet 2	33,333	30,000	53,333
Wet 3	11,111	16,667	28,889
Wet 4	10,000	22,222	15,556
Air 1	22,222	50,000	115,556
Air 2	15,556	10,000	33,333
Air 3	20,000	108,889	16,667
Air 4	18,888	75,556	26,667
Inc. 1	24,444	22,222	123,333
Inc. 2	28,889	63,333	31,111
Inc. 3	22,222	24,444	27,778
Inc. 4	46,667	30,000	26,667

All cultures successfully grew when a portion (< 0.025 g) was re-plated 1 month after the conclusion of the experiment; both the 24 °C and 38 °C treatment groups remained viable with visible hyphal growth when plated. No growth was seen when the liquified samples used for spore counting were plated; spores remained intact and visible but were sterilized at 24 h of 95 °C.

### Discussion

While chlamydospore production can be increased through optimization of growth media and supplementation (Sagüés et al. 2012), the results of this study indicate that further processing of fungal cultures such as challenging with dry conditions can increase the number of chlamydospores produced, increasing *Duddingtonia flagran*’s commercialization potential as a parasitic control agent. An added benefit to incorporating a drying technique for industrial application is the low cost. With the 24 °C “air-dried” and 38 °C “incubated” treatments yielding similar results, incubation chambers would be optional, allowing a producer to focus on time-to-dry versus incubation energy requirements for cost benefit analysis of producing enough spores to reach anthelmintic efficacy thresholds for feed additives.

One of the important outcomes of these study results concerns determining efficacy thresholds when using *D. flagrans* as an anti-parasitic feed additive. The observed effect of an increase in chlamydospore formation during drying should be taken into consideration when identifying the effective dosage in chlamydospores per gram if the feed additive is a dry supplement. Studies have shown a reduction in spore viability when passing through the digestive track of ruminants as high as 89.7% (Ojeda-Robertos et al. 2009), which means it is necessary to have a sufficient number of spores present in the feed to create the desired growth of *D. flagrans* in feces. Effective dosage has been observed in a variety of parasitic nematode host species such as goats (5 × 10^5^ chlamydospores/kg body weight (BW)) (Parauda et al. 2005), sheep (10^5^ chlamydospores/kg BW) (Peña et al. 2002), horses (2 × 10^6^ chlamydospores/kg BW) (Baudena et al. 2000), and even exotics such as giraffes and antelope (3 × 10^4^ chlamydospores/kg BW) (Young 2018), often measured in chlamydospores per kilogram bodyweight. If the number of chlamydospores being added to feed was measured in moist culture before a process of drying, the actual number of spores being fed (therefore true effective dosage) could be much higher than the measured amount: spore counts should be conducted post-drying to determine the dosage efficacy based on these study results.

Chlamydospore production may also be promoted using media supplements such as meso-inositol 0.5% to Sabouraud glucose agar or Tween20 for increased mycelium growth (Sagüés et al. 2012). Combined with post-growth drying to push these previously defined chlamydospore growth limits, these measures suggest a comprehensive strategy to maximize spore production.

A follow-up of general spore viability was done to demonstrate the drying did not render the spores non-viable. The result of all cultures from the 2 treatment groups and the control group remaining viable is not surprising since the spores survive that approximate temperature range when passing through a digestive tract and that *D. flagrans* chlamydospores have been shown to survive drying for as long as 7 years (Braga et al. 2011). While the viability of the dried culture spores demonstrate the robustness of this method, our dried treatment samples (8% moisture) were maintained at 37% and 55% humidity environments and were not exposed to extreme desiccation. Lower viability may result from vacuum desiccation or more extreme heat treatment. Additionally, specific spore percentage viability was not measured, only that < 0.025 g of the dried media (averaging approx. 16,000 spores) created visible mycelial growth when plated; percentage viability may be a consideration in future experimentation.

In conclusion, the drying of *Duddingtonia flagrans* cultures can significantly increase production of chlamydospores solving a limiting factor in the design of production pipelines for commercial use of this species as a parasitic control agent. The moisture content should also be taken into consideration in experimental design when measuring number of chlamydospores per gram in any feed additive as a fluctuation in this moisture level could change the number of spores being administered.
